# Supplementary figures and images for: Effects of TNFα receptor TNF-Rp55- or TNF-Rp75- deficiency on corneal neovascularization and lymphangiogenesis in the mouse
Source: PLoS One. 2021 Apr 9;16(4):e0245143. doi: 10.1371/journal.pone.0245143 (PMC8034740; doi:10.1371/journal.pone.0245143)

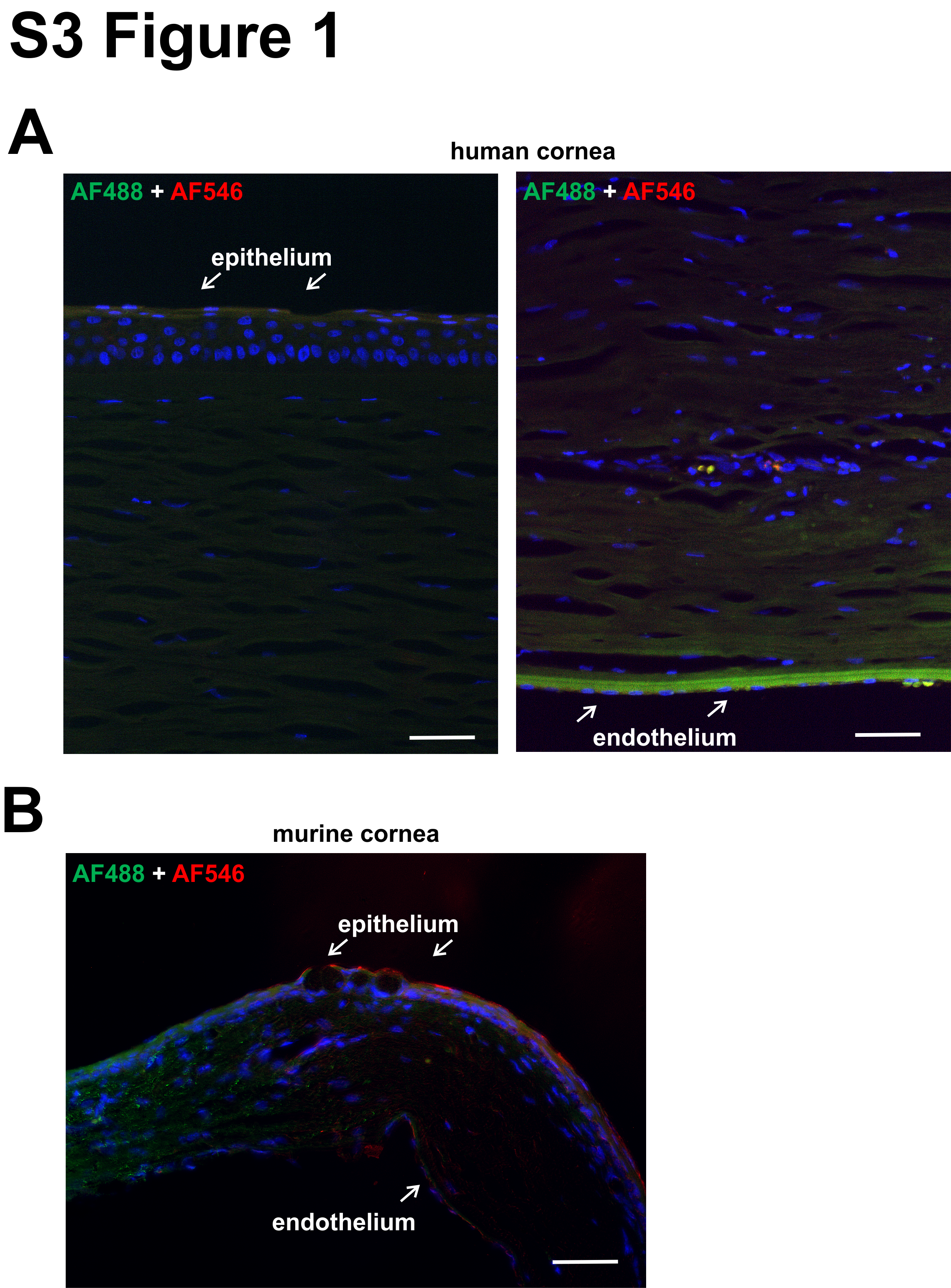

Supplement: S1 Fig — A (left side) depicts epithelium and stromal part of the cornea, while A (right side) depicts stroma and endothelium. Scale bars represent 100μm (A) or 50μm (B). (TIF) [file pone.0245143.s001.tif]

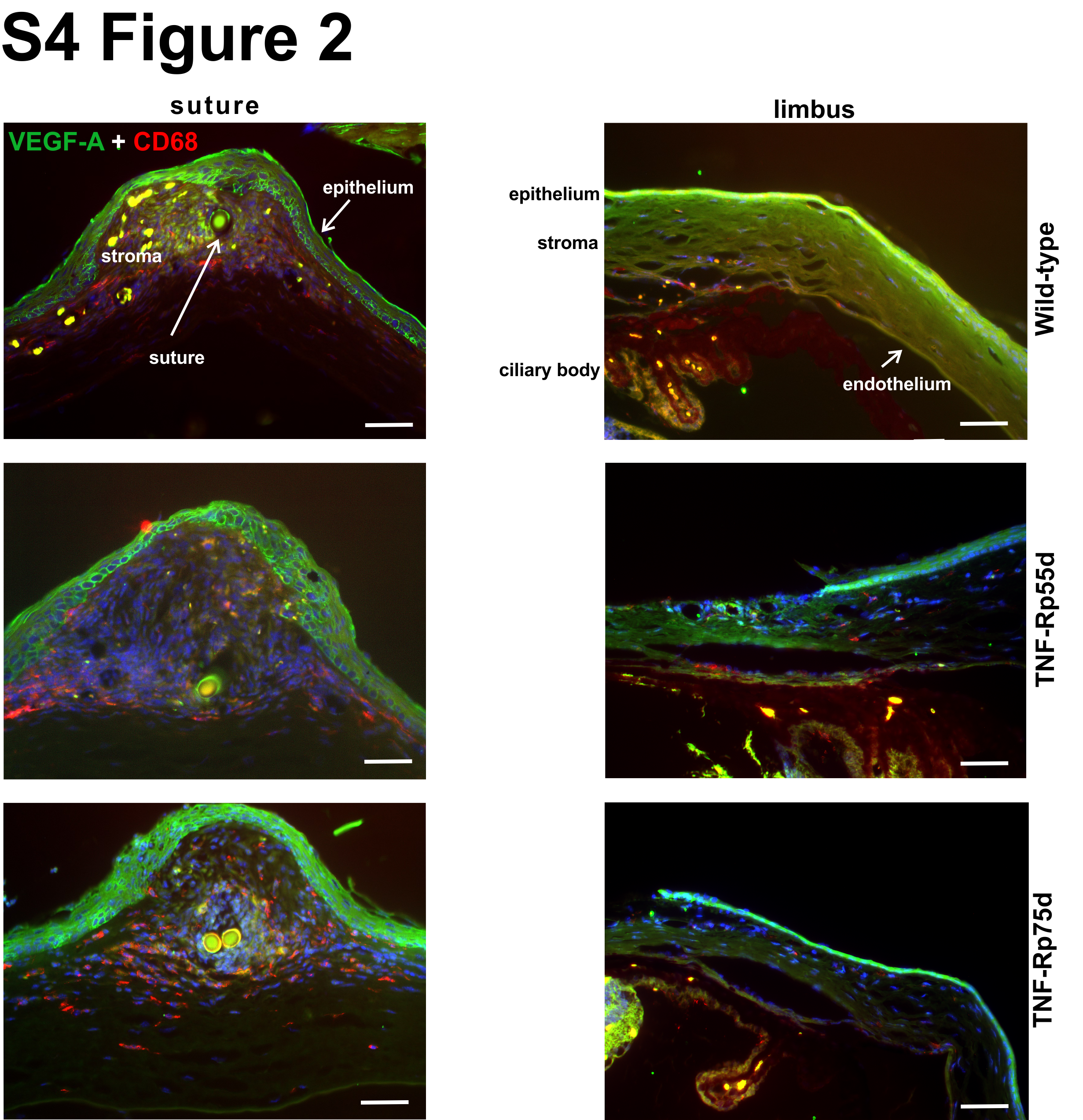

Supplement: S2 Fig — Immunohistochemical staining of corneal sections of sutured wild-type, TNF-Rp55d, and TNF-Rp75d mice in the suture and the limbus area using antibodies against VEGF-A (green) and CD68 (red). Nuclei were stained with DAPI. Scale bar represents 50 μm. (TIF) [file pone.0245143.s002.tif]

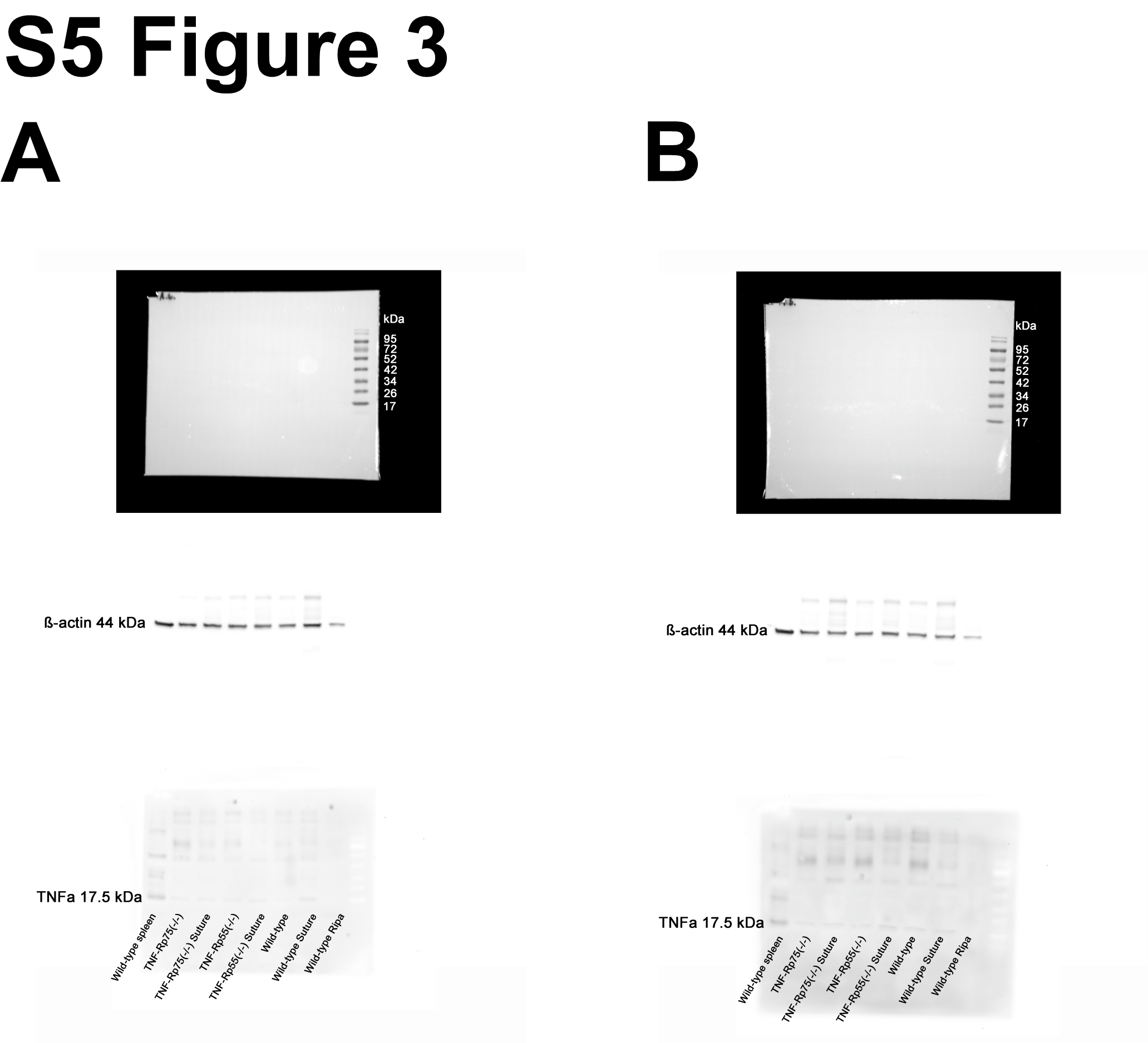

Supplement: S3 Fig — A, B: Uncropped representative images of Western blots used for the densitometric analysis in Fig 4C. All uncropped Western blots are shown in the Data repository. (TIF) [file pone.0245143.s003.tif]
