# Supplementary material for: Effects of TNFα receptor TNF-Rp55- or TNF-Rp75- deficiency on corneal neovascularization and lymphangiogenesis in the mouse
Source: PLoS One. 2021 Apr 9;16(4):e0245143. doi: 10.1371/journal.pone.0245143 (PMC8034740; doi:10.1371/journal.pone.0245143)
Supplement: S1 Table — (DOCX) [file pone.0245143.s004.docx]

Supplement Table 1: qPCR-Data

| **TNF-Rp55** | WT | | | TNF-Rp55 d | | | TNF-Rp75 d | | | |  |  |  |  |  |  |  |  |
| --- | --- | --- | --- | --- | --- | --- | --- | --- | --- | --- | --- | --- | --- | --- | --- | --- | --- | --- |
|  | Mean | SD | N | Mean | SD | N | Mean | SD | | N |  |  |  |  |  |  |  |  |
|  |  |  |  |  |  |  |  |  | |  |  |  |  |  |  |  |  |  |
| 8d | 1 | 0,152 | 2 | 0,347 | 0,039 | 3 | 0,797 | 0,151 | | 9 |  |  |  |  |  |  |  |  |
|  |  |  |  |  |  |  |  |  | |  |  |  |  |  |  |  |  |  |
|  |  |  |  |  |  |  |  |  | |  |  |  |  |  |  |  |  |  |
| **TNF-Rp75** | WT | | | TNF-p55 d | | | TNF-p75 d | | | |  |  |  |  |  |  |  |  |
|  | Mean | SD | N | Mean | SD | N | Mean | SD | | N |  |  |  |  |  |  |  |  |
|  |  |  |  |  |  |  |  |  | |  |  |  |  |  |  |  |  |  |
| 8d | 1 | 1,619 | 2 | 0,686 | 0,786 | 3 | 0,7 | 0,849 | | 4 |  |  |  |  |  |  |  |  |
|  |  |  |  |  |  |  |  |  | |  |  |  |  |  |  |  |  |  |
|  |  |  |  |  |  |  |  |  |  |  |  |  |  |  |  |  |  |  |
| **VEGF-A** | WT | | | WT suture | | | TNF-Rp55d | | | TNF-Rp55d suture | | | TNF-Rp75d | | | TNF-Rp75d suture | | |
|  | Mean | SD | N | Mean | SD | N | Mean | SD | N | Mean | SD | N | Mean | SD | N | Mean | SD | N |
| 3d | 1 | 0,050 | 3 | 2,83 | 0,255 | 3 | 1 | 0,051 | 3 | 2,197 | 0,479 | 3 | 1 | 0,045 | 3 | 1,567 | 0,722 | 3 |
| 8d | 1 | 0,023 | 2 | 1,442 | 0,160 | 2 | 1 | 0,140 | 2 | 1,662 | 0,187 | 2 | 1 | 0,052 | 2 | 1,924 | 0,081 | 2 |
| 14d | 1 | 0,100 | 3 | 2,156 | 1,031 | 3 | 1 | 0,094 | 3 | 1,896 | 0,826 | 3 | 1 | 0,072 | 3 | 1,543 | 0,387 | 3 |
|  |  |  |  |  |  |  |  |  |  |  |  |  |  |  |  |  |  |  |
| **VEGF-C** | WT | | | WT suture | | | TNF-Rp55d | | | TNF-Rp55 d suture | | | TNF-Rp75 d | | | TNF-Rp75 d suture | | |
|  | Mean | SD | N | Mean | SD | N | Mean | SD | N | Mean | SD | N | Mean | SD | N | Mean | SD | N |
| 3d | 1 | 0,061 | 3 | 0,707 | 0,293 | 3 | 1 | 0,046 | 3 | 0,551 | 0,180 | 3 | 1 | 0,042 | 3 | 0,673 | 0,398 | 3 |
| 8d | 1 | 0,044 | 3 | 1,015 | 0,206 | 3 | 1 | 0,087 | 3 | 0,780 | 0,238 | 2 | 1 | 0,042 | 3 | 0,980 | 0,256 | 3 |
| 14d | 1 | 0,082 | 3 | 0,46 | 0,182 | 3 | 1 | 0,056 | 3 | 0,999 | 0,236 | 3 | 1 | 0,129 | 3 | 1,367 | 0,519 | 3 |
|  |  |  |  |  |  |  |  |  |  |  |  |  |  |  |  |  |  |  |
| **Lyve-1** | WT |  |  | WT suture | | | TNF-Rp55d | | | TNF-Rp55 d suture | | | TNF-Rp75 d | | | TNF-Rp75 d suture | | |
|  | Mean | SD | N | Mean | SD | N | Mean | SD | N | Mean | SD | N | Mean | SD | N | Mean | SD | N |
| 3d | 1 | 0,079 | 3 | 1,062 | 0,819 | 3 | 1 | 0,075 | 3 | 0,976 | 0,632 | 3 | 1 | 0,039 | 3 | 0,704 | 0,223 | 3 |
| 8d | 1 | 0,073 | 2 | 1,226 | 0,052 | 2 | 1 | 0,098 | 3 | 0,986 | 0,440 | 3 | 1 | 0,089 | 3 | 1,356 | 0,440 | 3 |
| 14d | 1 | 0,072 | 3 | 0,542 | 0,331 | 3 | 1 | 0,058 | 3 | 1,408 | 0,457 | 3 | 1 | 0,057 | 3 | 1,517 | 0,197 | 3 |
|  |  |  |  |  |  |  |  |  |  |  |  |  |  |  |  |  |  |  |
| **TNF-a** | WT | | | WT suture | | | TNF-Rp55d | | | TNF-Rp55d suture | | | TNF-Rp75d | | | TNF-Rp75d suture | | |
|  | Mean | SD | N | Mean | SD | N | Mean | SD | N | Mean | SD | N | Mean | SD | N | Mean | SD | N |
| 3d | 1 | 0,109 | 3 | 0,965 | 0,580 | 3 | 1 | 0,057 | 3 | 0,596 | 0,242 | 3 | 1 | 0,055 | 3 | 0,510 | 0,219 | 3 |
| 8d | 1 | 0,043 | 2 | 0,337 | 0,080 | 2 | 1 | 0,033 | 3 | 1,191 | 0,082 | 2 | 1 | 0,026 | 3 | 0,522 | 0,200 | 3 |
| 14d | 1 | 0,088 | 3 | 0,308 | 0,026 | 3 | 1 | 0,059 | 3 | 0,463 | 0,217 | 3 | 1 | 0,054 | 3 | 1,202 | 0,763 | 3 |
