# Supplementary material for: Effects of TNFα receptor TNF-Rp55- or TNF-Rp75- deficiency on corneal neovascularization and lymphangiogenesis in the mouse
Source: PLoS One. 2021 Apr 9;16(4):e0245143. doi: 10.1371/journal.pone.0245143 (PMC8034740; doi:10.1371/journal.pone.0245143)
Supplement: S2 Table — (DOCX) [file pone.0245143.s005.docx]

Suppl Table 2: western blot

| **TNF-Rp55** | TNF-Rp55 d | | | TNF-Rp75 d | | | WT | | |
| --- | --- | --- | --- | --- | --- | --- | --- | --- | --- |
|  | Mean | SD | N | Mean | SD | N | Mean | SD | N |
|  |  |  |  |  |  |  |  |  |  |
| 8d | 0,338 | 0,1815 | 3 | 0,2105 | 0,0553 | 3 | 0,2916 | 0,1434 | 3 |
|  |  |  |  |  |  |  |  |  |  |
