# Supplementary material for: Effects of TNFα receptor TNF-Rp55- or TNF-Rp75- deficiency on corneal neovascularization and lymphangiogenesis in the mouse
Source: PLoS One. 2021 Apr 9;16(4):e0245143. doi: 10.1371/journal.pone.0245143 (PMC8034740; doi:10.1371/journal.pone.0245143)

Blots for Figure 1 F, G

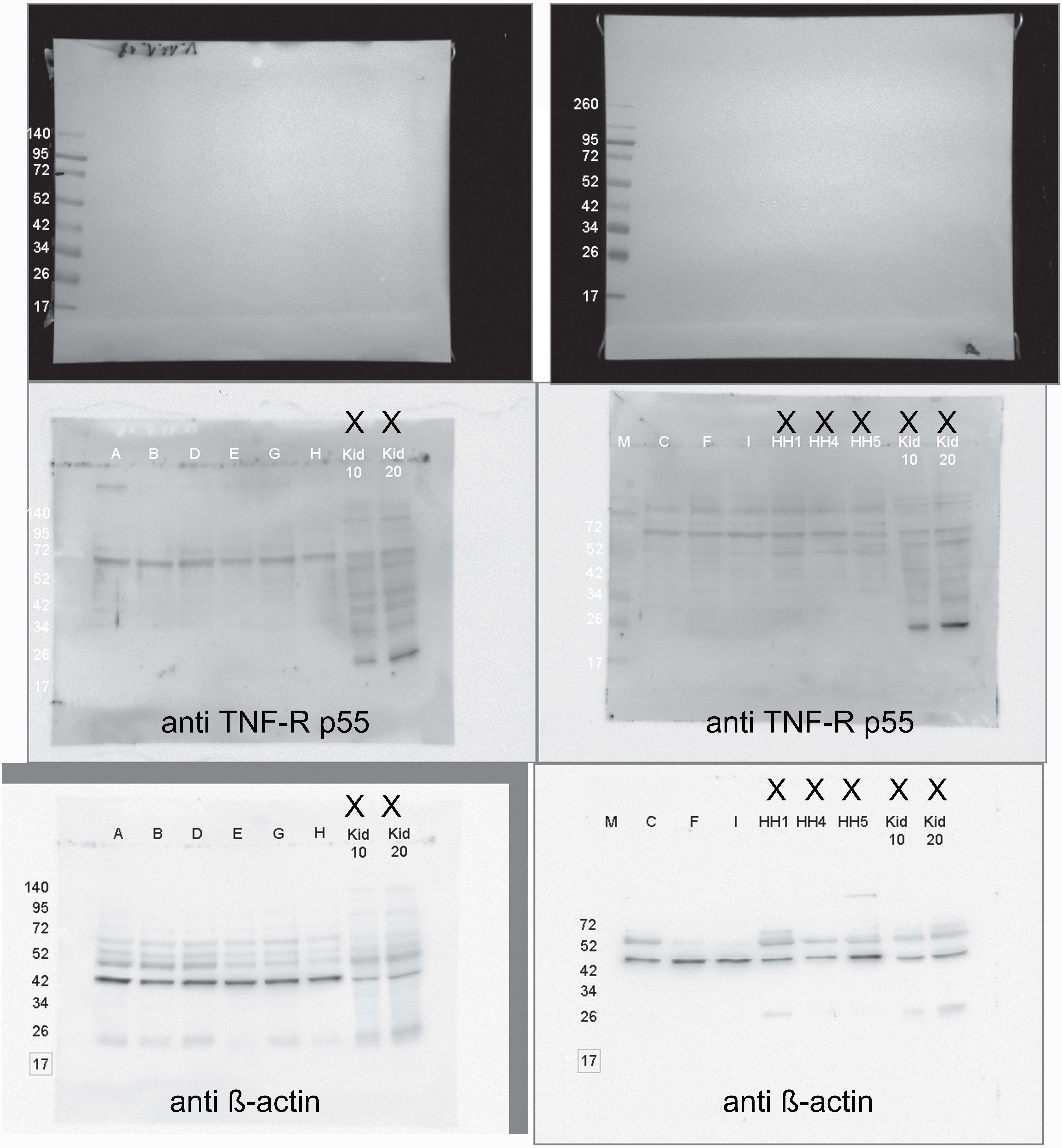

Blot 1 Blot 2

A, B, C: TNF-Rp55d; D, E, F: TNF-Rp75d; G, H, I: Wild-type

Blot 1 for Figure 4C

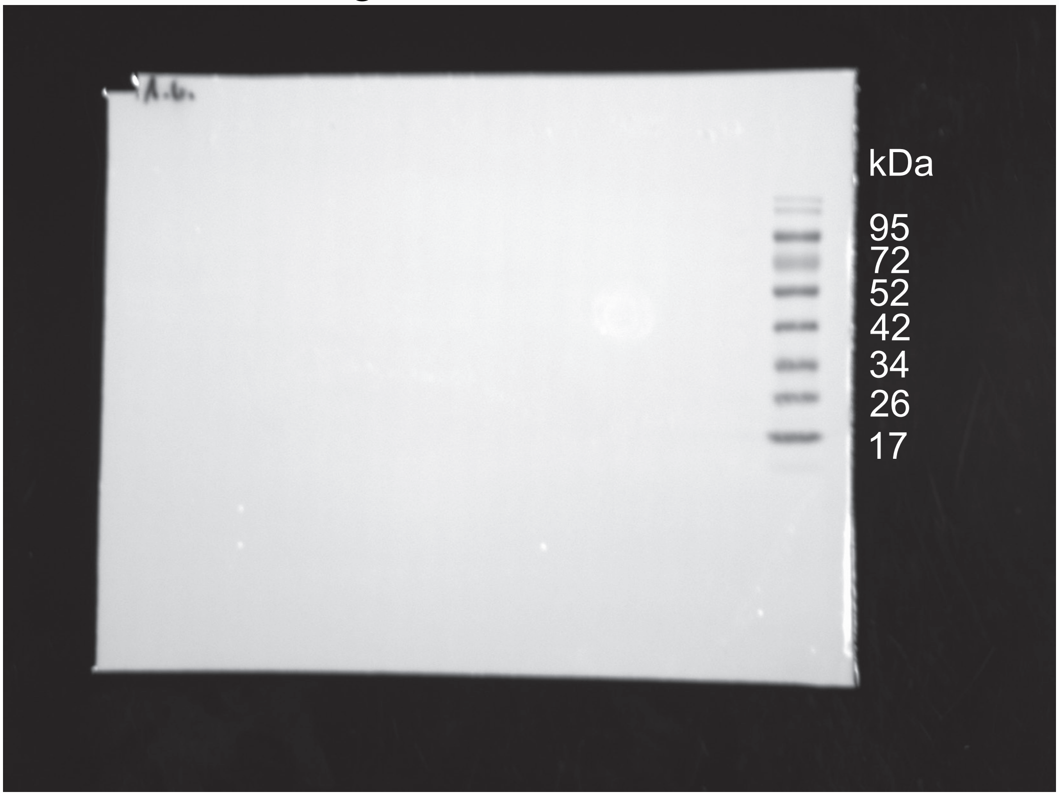

ß-actin 44 kDa

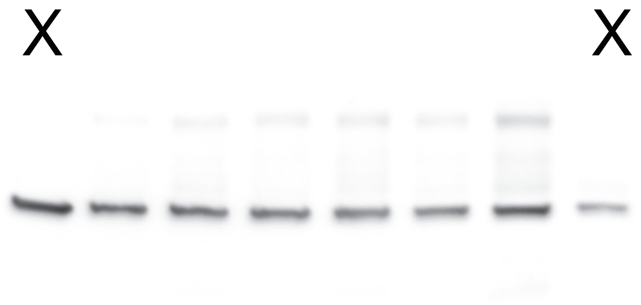

TNFa 17.5 kDa

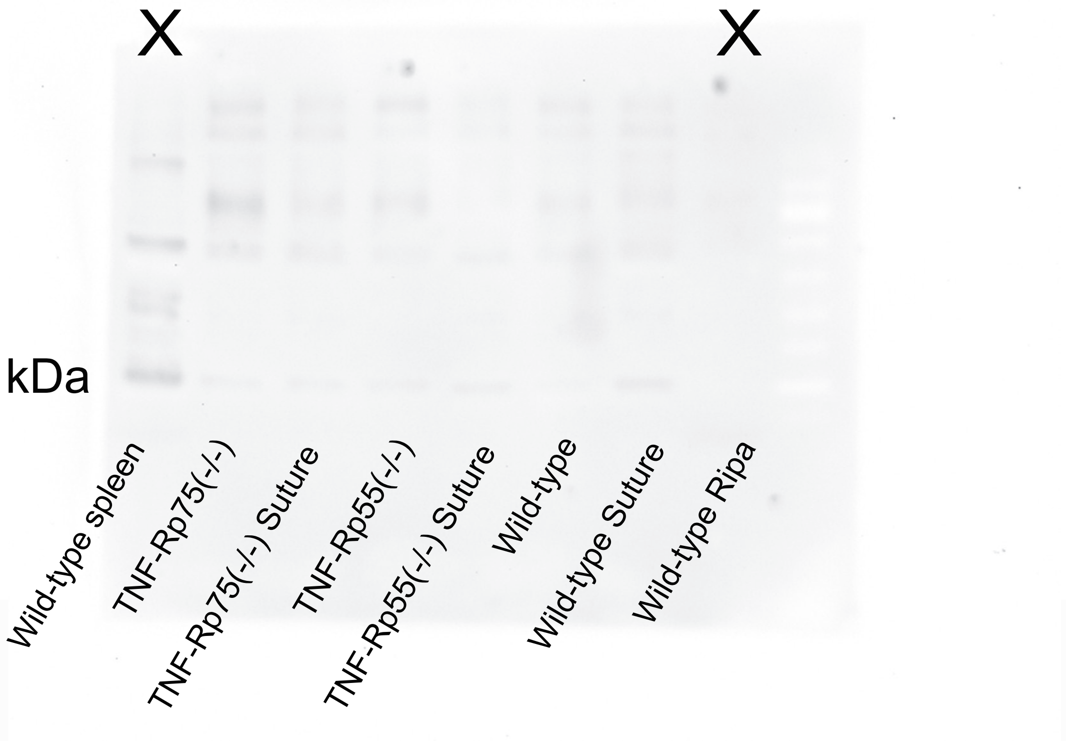

Blot 2 used for Figure 4C

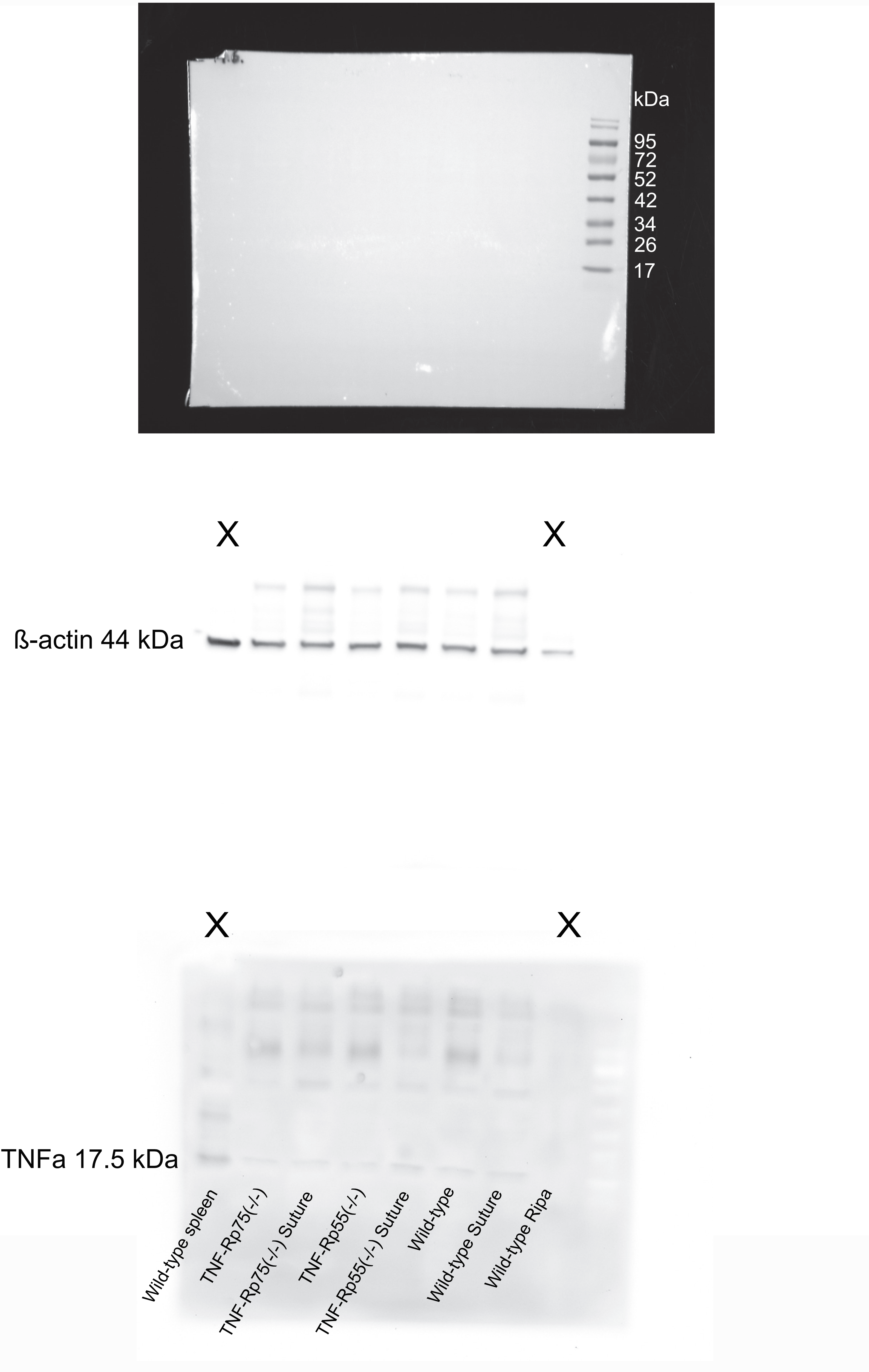

Blot 3 used for Fig 4B and C

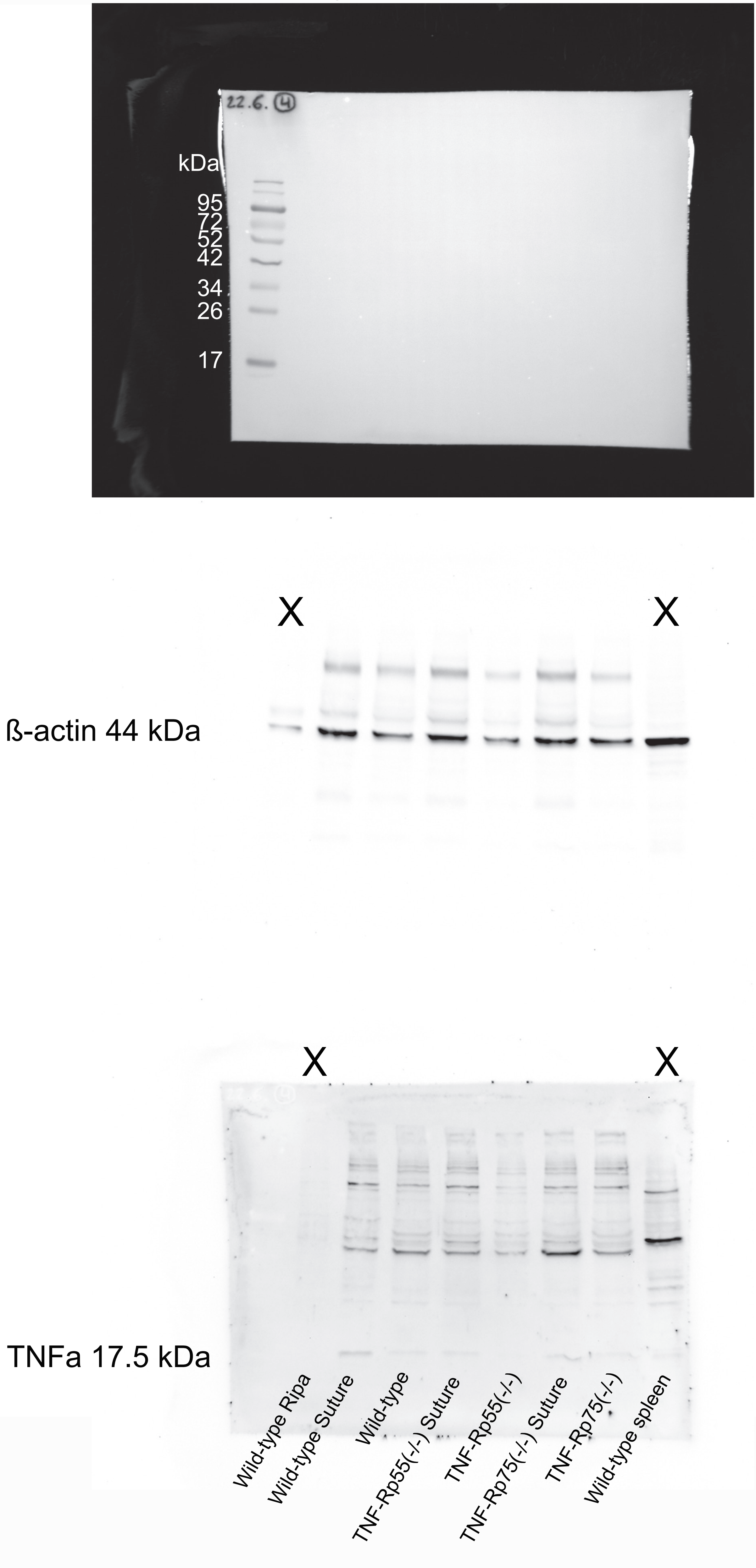

Blot 4 used for Figure 4C

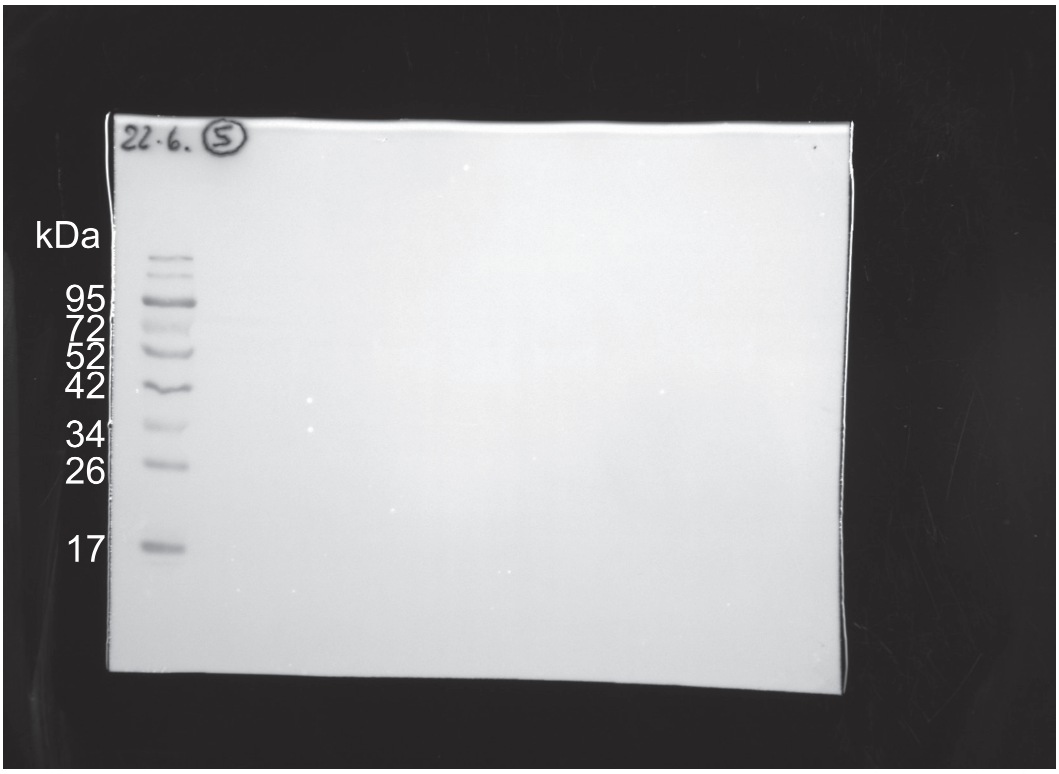

ß-actin 44 kDa

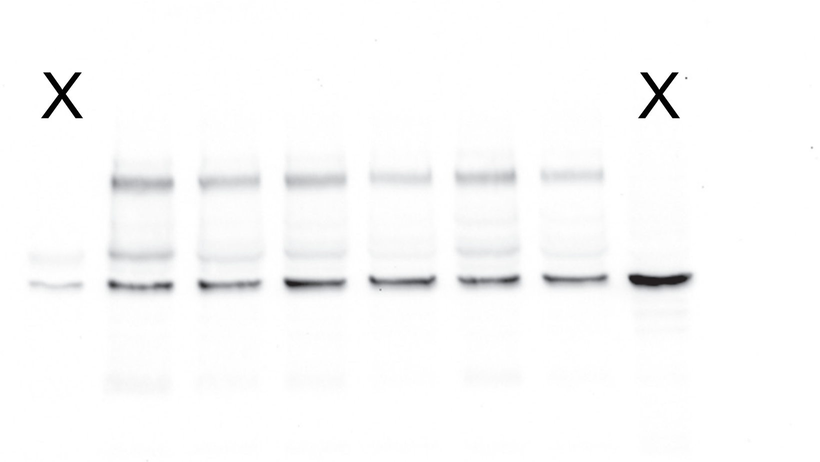

TNFa 17.5 kDa

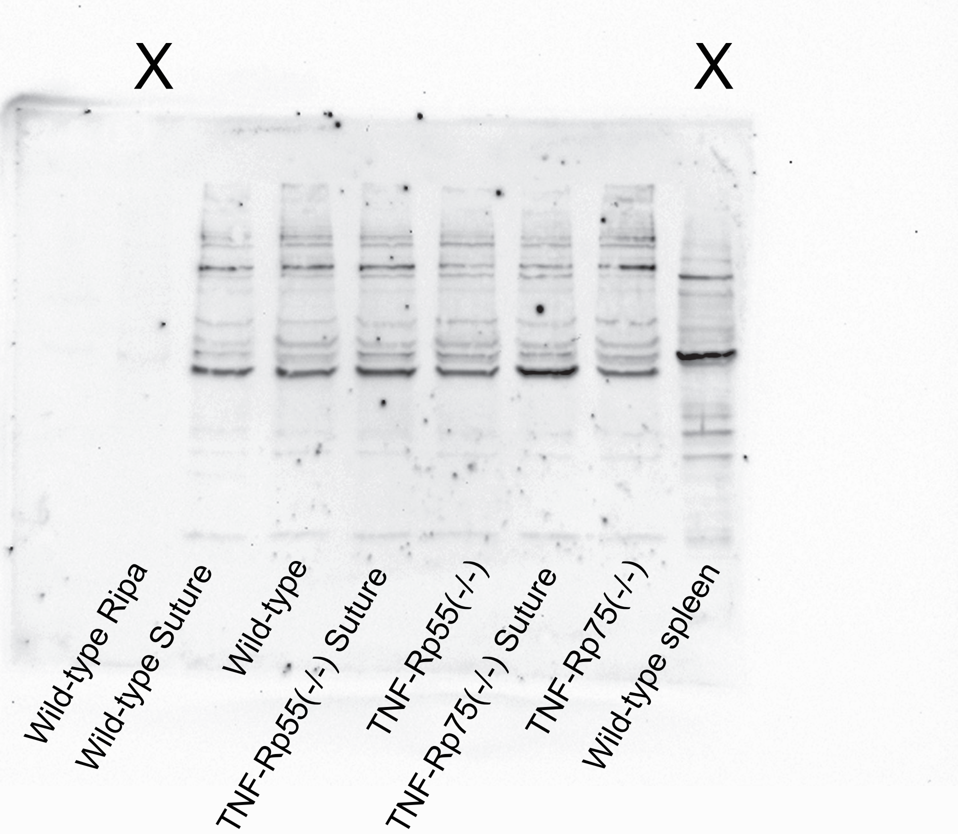

Supplement: S1 Raw images — (PDF) [file pone.0245143.s006.pdf]
