# Supplementary material for: Effects of TNFα receptor TNF-Rp55- or TNF-Rp75- deficiency on corneal neovascularization and lymphangiogenesis in the mouse
Source: PLoS One. 2021 Apr 9;16(4):e0245143. doi: 10.1371/journal.pone.0245143 (PMC8034740; doi:10.1371/journal.pone.0245143)
Supplement: Attachment — Submitted filename: Revision_TNFa_AnswerRevisionNRfinal.docx [file pone.0245143.s007.docx]

We thank the editorial board to give us the opportunity to improve our paper substantially according to the academic editor and reviewers’ comments.

We answer the academic editor and reviewer’s concerns point by point as follows.

**Journal requirements:**

1. Please ensure that your manuscript meets PLOS ONE's style requirements, including those for file naming. The PLOS ONE style templates can be found at https://journals.plos.org/plosone/s/file?id=wjVg/PLOSOne_formatting_sample_main_body.pdf and

https://journals.plos.org/plosone/s/file?id=ba62/PLOSOne_formatting_sample_title_authors_affiliations.pdf

**Answer:** We revised the manuscript according to the PLOS ONE`s style requirements.

2. To comply with PLOS ONE submissions requirements, in your Methods section, please provide additional information on the animal research and ensure you have included details on (a) methods of sacrifice, (b) methods of anesthesia and/or analgesia, and (c) efforts to alleviate suffering.

**Answer:** We included the details on (a) methods of sacrifice, (b) methods of anesthesia and/or analgesia, and (c) efforts to alleviate suffering.

“After suture placement, the mice were regularly examined by animal keepers. All efforts were made to minimize discomfort of the animals.”

“In brief, mice were deeply anesthetized by injection of ketamine/xylazine.”

“3, 8, 14 or 21 days after suture, mice were sacrificed by injection of ketamine/xylazine and subsequent cervical dislocation, and eyes were processed for further experiments.”

3. We note that you have stated that you will provide repository information for your data at acceptance. Should your manuscript be accepted for publication, we will hold it until you provide the relevant accession numbers or DOIs necessary to access your data. If you wish to make changes to your Data Availability statement, please describe these changes in your cover letter and we will update your Data Availability statement to reflect the information you provide.

**Answer:** All data are available at the open access database Zenodo using the following link (https://doi.org/10.5281/zenodo.4049178)

4. PLOS ONE now requires that authors provide the original uncropped and unadjusted images underlying all blot or gel results reported in a submission’s figures or Supporting Information files. This policy and the journal’s other requirements for blot/gel reporting and figure preparation are described in detail at https://journals.plos.org/plosone/s/figures#loc-blot-and-gel-reporting-requirements and https://journals.plos.org/plosone/s/figures#loc-preparing-figures-from-image-files. When you submit your revised manuscript, please ensure that your figures adhere fully to these guidelines and provide the original underlying images for all blot or gel data reported in your submission. See the following link for instructions on providing the original image data: https://journals.plos.org/plosone/s/figures#loc-original-images-for-blots-and-gels.

In your cover letter, please note whether your blot/gel image data are in Supporting Information or posted at a public data repository, provide the repository URL if relevant, and provide specific details as to which raw blot/gel images, if any, are not available. Email us at plosone@plos.org if you have any questions.

**Answer:** We added Figure S3 Fig depicting 2 representative uncropped blots. Furthermore, all blot data are available at a public data repository (https://doi.org/10.5281/zenodo.4049178).

S3 Fig

5. We note that you have included the phrase “data not shown” in your manuscript. Unfortunately, this does not meet our data sharing requirements. PLOS does not permit references to inaccessible data. We require that authors provide all relevant data within the paper, Supporting Information files, or in an acceptable, public repository. Please add a citation to support this phrase or upload the data that corresponds with these findings to a stable repository (such as Figshare or Dryad) and provide and URLs, DOIs, or accession numbers that may be used to access these data. Or, if the data are not a core part of the research being presented in your study, we ask that you remove the phrase that refers to these data.

**Answer:** Data of melting curve analysis are available at a public data repository (https://doi.org/10.5281/zenodo.4049178).

**Reviewer #1:**

**Q1:** In this study, Maier et al. investigate the potential role of TNFRs 1 and 2 in corneal neovascularization and lymphangiogenesis. The authors find a decrease in the vascularized area in TNFR-deficient animals as well as well as downregulation of TNF itself in their model. However, they conclude that TNF as well as its receptors do not play a significant role in the pathogenesis of neovascularization and lymphangiogenesis. The authors should consider moderating this statement and their interpretation.

**Answer:** We investigated the role of TNFα in the suture model at two levels: differential TNFα gene expression and aberration of TNF receptor function. TNFα expression remained unchanged after suture placement. Suture placement led to differential regulation of TNFα at the mRNA level, no significant changes were detectable in protein levels, though. No differences were detectable in TNFα comparing WT to mutants. Indeed, the reviewer is right that we found differences in vascularization (determined by CD31 coverage). The differences, however, were very subtle and only detectable at 1 time point (D14). The animal model should show that TNFα -signaling might play a role by increasing TNFα -sensitivity of participating cells at constant TNFα levels. Again, these data cannot provide evidence for a significant role of TNFα- signaling in the suture model.

Fig1

**Q2:** The data in figure 1 would be more convincing if a negative control was included. Since the authors have access to the TNFR-deficient mice, these samples should be included to demonstrate specificity of their antibodies.

**Answer:** We agree with the reviewer that we did not show a control for immunohistochemistry by using specimen from TNFR deficient tissue. For that we would have to use TNFR KO mice, lacking the expression of the respective gene by transgenic knocked down. In our case, however, we use KI models leading to lack of function of the respective proteins. Here, TNF-Rp55 and TNF-Rp75 are produced but their functional activity was switched off by the transgene. For more details how the KI mutations were created see answer to Q4. An isotype control, depicting the background staining of the secondary antibody, is included to the supplement (S1 Fig). We added arrows and text to ease the understanding of the figure

**Q3:** What was the reason for using two different antibodies (CD68 and F4/80) in these samples?

**Answer:** We used CD68 predominantly for human samples to detect macrophages and F4/80 in murine samples since they are more convincing to identify all macrophages in mice [1]. Unfortunately, F4/80 is only detectable in rodents.

**Q4:** It is not clear why the TNFR-deficient mice would show TNFR gene expression. The authors should validate their RT primers and repeat these experiments.

**Answer:** This is a misunderstanding of our model. We have to apologize that the current description was not clear enough. Thus, we now clarify the nature of the mutant mice, their genetic background and the functional consequences more precise. As already stated above, the TNFR deficiency is a functional one. The mice harbor a null mutation of the respective TNFR leading to an altered gene product that lacks the molecular function of the wild-type gene. The non-functional proteins themselves are expressed, however. The TNF-Rp55 null mutations were created by disruption of the coding sequence of the TNF-Rp55 gene at base pair 535 (corresponding to the cDNA sequence) by insertion of a neo gene [2]. This resulted in the transcription and transduction of a non-functional TNF-Rp55 protein. Pfeffer et al validated the mutation by an experiment that showed the inability of spleen cells of the TNF-Rp55^-/-^ mice to bind TNF-a
A similar strategy was pursued by Erickson et al creating the TNF-Rp75^-/-^ mice [3]. In TNF-Rp75^-/-^ mice challenged by LPS (that leads to shedding of the extracellular domain of TNF-Rp75), no soluble TNF-Rp75 could be detected in the serum.
We added the following sentence in the methods part to explain precisely the KI Model (p.5 Line 116ff:

“The mice harbor a null mutation of the respective TNFR leading to an altered gene product that lacks the molecular function of the wild-type gene. The non-functional proteins themselves are expressed. The TNF-Rp55 mutation was created by disruption of the coding sequence of the TNF-Rp55 gene by insertion of a neo gene (21). This resulted in the transcription and transduction of a non-functional TNF-Rp55 protein. A similar strategy was pursued by Erickson et al creating the TNF-Rp75d mice. A neomycin-resistance gene under control of the Pgk promoter20 was inserted in the second exon, which contains the signal peptide region of TNF-R2, resulting in the transcription and transduction of a non-functional TNF-Rp75 protein (22)”

**Q5:** Also, the western blot in figure 1F is inconclusive. Why is there no difference between wild type and knock out? Immunoblots for TNFR1 should also be included.

We validated the role of the TNFα signaling at the level of the TNFα -receptors. Figure 1F is an immunoblot showing the protein expression of TNF-Rp55 in *TNF-Rp55d*, *TNF-Rp75d* mice and wild-type littermates. As shown in bar chart 1G, there is no difference in protein expression of TNF-Rp55 among the groups. Especially TNF-Rp55 is not upregulated in the TNFrp-55d mice. Thus, we have convincing evidence, for the lack of compensatory upregulation of the TNFα-receptors expression in the KI-animals.

Fig2

**Q6:** CD31 positive/blood vessel area should be highlighted/indicated in figures 2B and 3C

**Answer:** We indicated the CD31/Lyve-1 covered area by arrows

Fig2 and Fig3

**Q7:** The data in figure 4 is inconclusive and is hard to interpret as is. The quality of the immunoblot is rather low. The authors could also consider evaluating the function of TNF using an in vitro system. This would further strengthen interpretation and conclusion.

**Answer:** We tried to evaluate the role of TNFα signaling in suture induced neovascularization and lymphangiogenesis at several levels. We analyzed gene expression and protein expression of TNFα and its receptors in wild-type and *TNF-Rp55d* or *TNF-Rp75d* mice respectively and could not find reliable evidence. Thus, taken our results from animal experiments, in vitro experiments would not add valuable data to the manuscript. More complex signaling cascades that involve many different cell types and structures, an environment that is impossible to mimic in vitro might help to explain the pathogenesis of suture induced alterations in the animals.

The protein concentration of TNF-α in the cornea is very low, also compared to other, mainly structural proteins that are expressed abundantly in the cornea. We provide an uncropped, unedited version of the original blot (see S3Fig). Unfortunately we are not able to substantially improve the quality of the signal. Even precipitation of TNFα did not improve the visibility of the TNF-α band.

Minor comments:

**Q8:** The title is confusing and should be revised. The authors study the effects of deficiency rather than malfunction.

**Answer:** We changed the title to “Effects of TNFα *receptors* TNF-Rp55 or TNF-Rp75- deficiency on corneal neovascularization and lymphangiogenesis in the mouse.

**Q9:** The overall quality of the figure-PDF file is low. High resolution images and unified labels (font + size) should be provided with the revised manuscript.

**Answer:** The reviewer is right; the images in the current version are of poor quality/resolution. We significantly improved the resolution of the images and unified all the labels. We think that the current version is more appropriate now.

**Reviewer #2:** It is pleasing to read the report by Dr. Maier and colleagues in which the group tested the relative efficacy of two TNF receptor deletions on vascularization following corneal injury; the reporting of minimal or negative results is not done often enough and this finding herein is important. The mouse experiments and data are sound, with no problems in their presentation, interpretation, or subsequent discussion. There is some concern with the human data: it is very hard from the way the imaging studies were performed to claim co-localization or lack thereof from any particular cell type (the imaging problem exists with the mouse tissue as well). Since this paper is not presenting a tissue-specific deletion of either Rp55 or Rp75, it's relative localization is not critically important to the findings. Being purposefully vague might be the safest route, claiming that protein is expressed and 'does not appear' to localize with macrophages... the epithelium is usually 'hot' for fluorescence so that claim is questionable and S3 and S4 are not convincing (and lack proper labels and legends).

**Answer:** We thank the reviewer for his interest in the manuscript and we are thankful for the comments to improve the manuscript. We know that the results of co-localization analysis on sagittal sections have to be handled carefully. We agree with the Reviewer to state our conclusion more vaguely. Thus, we rephrased the sentence on p.9 line 228f. It is less speculative now by only stating “We also detected macrophages by CD68 staining, the receptors and macrophages, however, do not appear to co-localize.”

We renewed the negative controls by more convincing ones and added labels to S1 Fig (S3)/S2 Fig (S4) to ease the understanding at a glance.

**Reviewer #3:** In this manuscript, the authors characterized corneal neovascularization and lymphangiogenesis in the TNF-Rp55 or TNF-Rp75 KO mice. They concluded that in the suture-induced mouse model, TNFα and its ligands TNF-Rp55 and TNF-Rp75
do not play a significant role in corneal neovascularization and lymphangiogenesis. However, the data showed that a reduction of neovascularization in these KO mice compared with WT at day 14 and reduced lymphangiogenesis in TNF-Rp75 KO mice. This suggests that TNF signaling has a role in these two processes.

**Answer:** Indeed, the reviewer is right that we found differences in vascularization and lymphangiogenesis (determined by CD31 or LYVE-1 coverage). The differences, however, were very subtle and only detectable at 1 time point (D14). These subtle differences might be statistically significant but not biologically relevant: The exact values for CD31 coverage on day 14 are: WT 15.6% ± 2.6%, *TNF-Rp55d* 12.4% ± 2.6% and *TNF-Rp75d* 9.6% ± 0.8%; for LYVE-1 coverage WT 9.7% ± 1.3%, *TNF-Rp55d* 8.4% ± 1.9%, *TNF-Rp75d* 7.2% ± 2.2%. The animal model should show that TNFα -signaling might play a role by increasing TNFα -sensitivity of participating cells at constant TNFα levels. Again, these data cannot provide evidence for a significant role of TNFα- signaling in the suture model.

Other comments:

**Q1:** In most experiments, how many mice were used is unclear.

**Answer:** we provide numbers for all individual figures now in the figure legend and the supplement (S1 Table/S2 Table for WB and PCR) as well as an overview of the total amount of animals in the methods part. We added this sentence (p5. Lines 127-128): “A total number of 47 TNF-Rp55d mice, and 47 TNF-Rp75d mice and 48 wild-type mice were used for this study.”

**Q2:** The authors only explored the expression of 3 genes that are related to neovascularization and lymphangiogenesis. Since many genes involve in regulation of neovascularization and lymphangiogenesis, the authors can't make a conclusion that TNFα and its ligands TNF-Rp55 and TNF-Rp75 do not play a significant role.

**Answer:** The reviewer is right that we only focused on few markers of neovascularization and lymphangiogenesis. The scope of the study, however, was to assess the differences in standard markers for neovascularization and lymphangiogenesis comparing to different KI models and WT before and after suture placement. We consider VEGF-A/VEGF-C and LYVE-1 as very robust and established markers for these 2 events. A detailed analysis of plenty of different angiogenesis markers like angiopoietin-1 angiopoietin-2, Tie-1, or endothelial cell adhesion molecules like VE-cadherin, PECAM-1, or several integrins would be out of the scope of the manuscript [4]. Furthermore, the take home message of the paper might not benefit from this extension of the analysis due to several problems, e.g. different time-points of regulation and low expression levels that we already suffered from with our rather robust read-out. Taken together, we did not find differential expression of TNFα after suture placement nor any conclusive effects at the level of the TNF-receptors, respectively.

**Q3:** TNF-a is an important cytokine in regulating corneal inflammation, neovascularization and lymphangiogenesis. In this study, a suture-induced corneal neovascularization model was used to identify the effect of TNF-a in corneal neovascularization and lymphangiogenesis. The model is so mild, many of the immunological cells and cytokines were not found significantly changed in this model. So that’s possible that you can’t observe much effects of TNF-a in this situation. Also, the suture-induced corneal neovascularization is not suitable to mimic the clinical scenario of corneal graft rejection. As we know, the corneal graft rejection is reasoned by exogenous antigen stimulation, which is not similar to the suture induced corneal neovascularization.

**Answer:** Indeed the reviewer is right that the suture-induced corneal neovascularization is not the ideal mouse-model to analyze corneal graft rejection, but it mimics the clinical scenario of a high-risk situation for a corneal graft rejection after keratoplasty. Compared to the alkali burn induced corneal neovascularization, the suture-induced model has a number of advantages especially studying the corneal lymphangiogenesis and associated prolymphangiogenic cytokines [5]. First, the sutures are more reproducible than the alkali burns on the cornea. Suturing only causes localized epithelial loss and inflammatory infiltration between the suture and the limbus, but chemical burns deplete the whole epithelial layer of the central cornea and cause strong cellular infiltration of the whole cornea [6]. Therefore, the suture-induced model mimics the clinical scenario of mild corneal lesions better than the alkali burn induced model. Fortunately, chemical burns in patients have become less common.

We clarified this in the discussion part, deleted the chapter about corneal graft rejection and added the following paragraph (p13, lines 363ff).

“Second, it is more suitable to analyse lymphangiogenesis and associated secretion of prolymphangiogenic cytokines [5]. The induced neovascularisation and lymphangiogenesis mimic the clinical scenario of high-risk situation for a corneal graft rejection after keratoplasty and thus it has more translational impact than the models with severe damage and reduced stimulation of the lymphangiogenesis. Suturing only cause localized epithelial loss and inflammatory infiltration between the suture and the limbus, but chemical burns deplete the whole epithelial layer of the central cornea and cause strong cellular infiltration of the whole cornea [6].”

**Q4:** A group of non-injured mice need to be added in each experiment. The authors should identify the expression of TNF-a has been increased in your models. Otherwise, there is no point to investigate the roles of TNF-a and its receptors in this model.

**Answer:** The reviewer rises two important questions: the lack of an appropriate control and the absence of a significant TNFα increase upon suture placement.

We have an inert control: only one eye was sutured, the counter-eye served as control. We added a sentence in the method part to highlight the presence of an appropriate control (p6 lines 133f.). The fact that TNFα was not significantly increased in our model does not question our conclusions. First, it might be an issue of the perfect time point that we did not find increased TNFα protein expression. We only checked Day 14 after suture placement. Theoretically, the receptor could also become more sensitive to TNFα at the same concentration by time. We checked not only for TNFα expression, but also TNFα receptor expression as well as function. Thus, we can safely state that TNFα does not play a role in this model. The TNFα increase alone is not necessary or sufficient.

**Reviewer #4:** The authors show that TNF-α and its receptors are not required for neovascularization and lymphangiogenesis in corneal suture model in mice. The data presented in Fig1 E, F and G indicates that the authors are working with mice where the TNFR receptors are not knocked and thus cannot make any claim on the role of TNFR in angiogenesis and lymphangiogenesis. I have to recommend rejecting the paper in its current form.

The following issues need to be addressed.

**Q1:** The quality of tissue in immunofluorescence images Fig1A and S3 and resolution of image is very poor and cannot be interpreted. It is unclear what region of the eye is shown in Fig1A although from result section it appears to be cornea. There is no indication of where TNFα receptor are localizing in the cornea. Please indicate where the receptors are localizing.

**Answer:** We agree with the reviewer that the resolution of the images was very bad in the draft of our manuscript. We addressed this issue and we now provide significantly better images in S1 Fig (S3) and S2 Fig (S4). We added arrows to depict structures/ staining and we added several remarks in the figure legends to improve understanding.

**Q2:** Fig 1B suffers from the same issue as Fig1 A. The tissue section is of extremely poor quality. The limbus region is suspect. Please show a phase image so one might see the iridiocorneal angle. The labels in B and C are confusing is label for C on left makes no sense to the reader. Should it not be cornea? If B and C are from the suture experiment (says Mouse + Suture on the right of panels) where are the controls or sham eyes?

**Answer:** We agree with the reviewer that the images in Fig1B are lacking the iridiocorneal angle. We display a bigger detail of the images now showing the iridiocorneal to ensure the localization of the section (at the limbus corneae). Furthermore, we are very sorry for the confusion induced by the labelling. All images in Fig1B and C are from WT mice that were sutured. Fig1B illustrates expression of F4/80 and TNFRp55 (left) and TNFrp75 (right) in the limbus area whereas Fig1C shows the expression of F4/80 and TNFRp55 (left) and TNFRp75 (right) in the suture area. We improved the labelling and the figure legends to ease the understanding for the reader here.

**Q3:** Why is TNFR2 75KDa not reduced in the TNFR2 knockout mouse in Fig 1D? Please reword Line 223-227 “The mRNA expression of TNFRp55 in the TNF-RP55d mice, however, is significantly reduced (Fig.1D, E) and the functionality of the receptors is disturbed. There is no evidence for differential regulation of the other receptor in the TNF-Rpd animals, neither on mRNA nor on protein level (Fig.1D, E,F, G, S1 Table 1) as is very confusing.

**Answer:** As explained above the mutation in TNFR deficient mice does not lead to a lack of expression, thus, mRNA expression of TNFR2 does not have to be reduced in TNF-Rp75d mice. The explanation is as follows: As already stated above, the TNFR deficiency is a functional one. The mice harbor a null mutation of the respective TNFR leading to an altered gene product that lacks the molecular function of the wild-type gene. The non-functional proteins themselves, however, are expressed. The TNF-Rp55 null mutations were created by disruption of the coding sequence of the TNF-Rp55 gene at base pair 535 (corresponding to the cDNA sequence) by insertion of a neo gene [2]. This resulted in the transcription and transduction of a non-functional TNF-Rp55 protein. Pfeffer et al validated the mutation by an experiment that showed the inability of spleen cells of the TNF-Rp55^-/-^ mice to bind TNFα. Erickson et al, creating the TNF-Rp75-/- mice, pursued a similar strategy [3]. In TNF-Rp75^-/-^ mice challenged by LPS (that leads to shedding of the extracellular domain of TNF-Rp75), no soluble TNF-Rp75 could be detected in the serum. Furthermore, we rephrased the sentence p.9 lines 234-237 accordingly:” The mRNA expression of TNFRp55 in the *TNF-RP55d* mice, however, is significantly reduced (Fig1D, E). There is no evidence for differential regulation of TNF-Rp75 in *TNF-Rp55d* animals or vice versa, neither on mRNA nor on protein level (Fig1D, E, F, G, S1 Table)

**Q4:** Fig 1F and G leads to question the entire premise of the paper- it seems TNFR1 protein is present in mutant hence the authors are working with essentially wt mice?? What is the state of the TNFR2 mutant mice? Does it still express TNFR2 protein? Please validate mice before doing the suture experiments. Please show a western blot showing the protein levels of TNFR1 and TNFR2 in wt and respective mutant mice.

**Answer:** The reviewer rises several important questions. First, the expression of TNF-Rp55 in *TNF-Rp55d* mice. As already stated in the answer to Q4 (Reviewer1), the mice harbor null-mutations of the respective genes leading to a malfunction of the receptors and not a lack of expression. Second, the reviewer asks for WB analysis of TNFR1 and TNFR2 expression in the different KI-models/WT mice. Figure 1F depicts an immunoblot showing the protein expression of TNF-Rp55 in *TNF-Rp55d* mice, *TNF-Rp75*d mice and wild-type littermates. As shown in bar chart 1G there is no difference in protein expression of TNF-Rp55 among the groups. Unfortunately, we cannot provide a WB of TNF-Rp75 since the antibody did not show a proper signal in WB in several trials.

**Q5:** Also need to this experiment with the double knockout mice given TNFα knockout seems to exacerbate neovascularization (Fujita 2007).

**Answer:** Thank you for the recommendation. The TNFα knockout mouse model is a completely different approach compared to our KI models. To evaluate our hypothesis, we however, decided to investigate initially whether one receptor is upregulated when the other is deficient. However, TNF-Rp55 is not upregulated in the TNF-Rp75d mice. Thus, we have convincing evidence, for the lack of compensatory upregulation of the TNFα-receptors expression in the KI-animals on both gene expression and protein expression level. Additionally, TNFα expression remained unchanged after suture placement. Suture placement led to differential regulation of TNFα at the mRNA level, no significant changes were detectable in protein levels, though. No differences were detectable in TNFα comparing WT to mutants. Therefore, we do not expect any influence of TNFα in a double KI or KO model.

**Q6:** The figures all seem to be of poor resolution making it hard to zoom in on panels to see detail.

**Answer:** As already stated above, we substantially improved the resolution of all images and headings.

**REFERENCES**

1.	Khazen W, M'Bika J P, Tomkiewicz C, Benelli C, Chany C, Achour A, et al. Expression of macrophage-selective markers in human and rodent adipocytes. FEBS Lett. 2005;579(25):5631-4. Epub 2005/10/11. doi: 10.1016/j.febslet.2005.09.032. PubMed PMID: 16213494.

2.	Pfeffer K, Matsuyama T, Kundig TM, Wakeham A, Kishihara K, Shahinian A, et al. Mice deficient for the 55 kd tumor necrosis factor receptor are resistant to endotoxic shock, yet succumb to L. monocytogenes infection. Cell. 1993;73(3):457-67. Epub 1993/05/07. doi: 10.1016/0092-8674(93)90134-c. PubMed PMID: 8387893.

3.	Erickson SL, de Sauvage FJ, Kikly K, Carver-Moore K, Pitts-Meek S, Gillett N, et al. Decreased sensitivity to tumour-necrosis factor but normal T-cell development in TNF receptor-2-deficient mice. Nature. 1994;372(6506):560-3. Epub 1994/12/08. doi: 10.1038/372560a0. PubMed PMID: 7990930.

4.	Shih SC, Robinson GS, Perruzzi CA, Calvo A, Desai K, Green JE, et al. Molecular profiling of angiogenesis markers. The American journal of pathology. 2002;161(1):35-41. Epub 2002/07/11. doi: 10.1016/S0002-9440(10)64154-5. PubMed PMID: 12107087; PubMed Central PMCID: PMCPMC1850687.

5.	Giacomini C, Ferrari G, Bignami F, Rama P. Alkali burn versus suture-induced corneal neovascularization in C57BL/6 mice: an overview of two common animal models of corneal neovascularization. Exp Eye Res. 2014;121:1-4. Epub 2014/02/25. doi: 10.1016/j.exer.2014.02.005. PubMed PMID: 24560796.

6.	Jia C, Zhu W, Ren S, Xi H, Li S, Wang Y. Comparison of genome-wide gene expression in suture- and alkali burn-induced murine corneal neovascularization. Molecular vision. 2011;17:2386-99. Epub 2011/09/17. PubMed PMID: 21921991; PubMed Central PMCID: PMCPMC3171500.
