# Supplementary material for: Effects of TNFα receptor TNF-Rp55- or TNF-Rp75- deficiency on corneal neovascularization and lymphangiogenesis in the mouse
Source: PLoS One. 2021 Apr 9;16(4):e0245143. doi: 10.1371/journal.pone.0245143 (PMC8034740; doi:10.1371/journal.pone.0245143)
Supplement: Attachment — Submitted filename: TNFa_rebuttal2_finalNR.docx [file pone.0245143.s008.docx]

We thank the editorial board to give us the opportunity to improve our paper substantially according to the reviewers’ comments.

We answer the reviewer’s concerns point by point as follows.

Reviewer #1: The authors have improved the manuscript and addressed some of my concerns. However, there are fundamental discrepancies with previous literature that need to be explained prior to publication in PLOS ONE.

**Q1:** B6.129-Tnfrsf1atm1Mak/J, Jackson lab stock 002818 and B6.129S2-Tnfrsf1btm1Mwm/J, Jackson lab stock 002620 are targeted knockout animal strains and do not express TNFRp55 or TNFRp75 respectively.
These are conventional/“traditional” knockout mouse models that were generated in the Mid 90’s using a neomycin resistance gene flanked by homology arms to disrupt gene expression. Although the authors are correct that, in these studies, specific functional assays were used to confirm that the respective genes were no longer functional, this approach leads to a knockout of the targeted gene and not to a mutant form of the targeted gene.
In particular, while the authors indicate that the targeted approach by Pfeffer et al. leads to “the transcription and transduction of a non-functional TNF-Rp55 protein”, Pfeffer et al. generated mice that lack expression of TNFRp55. Similarly, Erickson et al. created mice that do not express TNFRp75. These 2 mouse lines are well-established null/knockout/deficient mouse models that have been utilized extensively. In addition, the knockout strategy is well-described in these two published studies.
With this in mind, the following questions still need to be addressed in a revised manuscript.

We thank the reviewer for his thoroughness concerning the knock-in models. We still think there is a massive misunderstanding.  We want to emphasize that B6.129-Tnfrsf1atm1Mak/J, Jackson lab stock 002818 and B6.129S2-Tnfrsf1btm1Mwm/J, Jackson lab stock 002620 lead to the transcription /translation of a truncated protein without proper function and are not KO-mutations in the sense that no mRNA and no protein at all are produced.

It is well known that the classic knockout models produced in the 90s are sometimes not full knockouts in a sense that the mRNA is truncated behind the neomycin cassette and the protein’s length corresponds with nucleotide sequence before the neomycin cassette. In some cases, mRNAS from more distal parts of the gene are produced and lead to a protein that represents a C-terminal part of the protein. Normally in the papers that introduce the model demonstrate the knockout most of the time by mRNA techniques for nucleotide sequences close to neomycin cassette. Thus, we checked this possibility in our study.

As the referee brought up this point in the sense to use *TNFRp55d* mice as antibody control for the anti-TNFR1 antibody we focus here in our response onto this mouse strain. The evidence comes from our PCR experiments where we show TNFRp55 expression in *TNFRp55d* mice, when using a primer that binds to nucleotide position 934-957 (F)/1085-1065 (R).

According to the information from Jackson about this strain, the neo cassette is inserted at nucleotide position 535. Using a primer pair that binds to nucleotide 500-520 (F) and 714-733 R or 481-501 (F) and 695-715 R, respectively did not lead to a specific amplification product in *TNFRp55*d mice, whereas in WT and *TNFRp75d* it did.

Figure1: mRNA expression of TNFRp55 in WT, *TNFrp55d* and *TNFRp75d*. Amplification product size: 234bp (left), 235bp (middle). GAPDH (190bp, right) Served as housekeeping gene. WT P17 O2 retina served as a murine control.

Along with this the immunogen for generating the antibody, we used for IHC and WB (CAT#: AP06465PU-N), Origene is a synthetic peptide, corresponding to the amino acids 370-420 of the human TNF-R1, and corresponding to the C-terminal end of the antibody. Therefore, we cannot use the *TNFRp55d* mice as an antibody control.

Thus, we conclude, that it is very likely that TNFRp55 is expressed as a truncated and non-functional protein in B6.129-Tnfrsf1atm1Mak/J.

We base this conclusion both on our experimental evidence and the data from Pfeffer and Erickson.

Our results presented in the manuscript are further substantiated by the new evidence derived from specific mRNA expression in *TNFRp55d* using primers beyond the neo-cassette and the use of C-terminal amino acids as immunogen.

All the following questions (Q2-Q5) address the same issue: the expression of TNFRp55 in T*NFRp55d* mice. Thus, for answering the questions we refer to the answer to Q1.

**Q2**: The data in figure 1 would be more convincing if a negative control was included. Since
the authors have access to the TNFR-deficient mice, these samples should be included to
demonstrate specificity of their antibodies.

Not feasible due to truncated protein and antibody binding at the C-terminal end.

**Q4:** It is not clear why the TNFR-deficient mice would show TNFR gene expression. The
authors should validate their RT primers and repeat these experiments.

TNF primers validated see above.

**Q5:** Also, the western blot in figure 1F is inconclusive. Why is there no difference between wild type and knock out? Immunoblots for TNFR1 should also be included.

See above; immunoblot for TNFR1. The western blot shows therefore the truncated protein in the TNFR1-KO, the full protein in the TNFR2 und WT probe. It shows that the TNFR2 knockout does not lead to a compensatory upregulation of the TNFR1.

Reviewer #2: Not sure why the labels on the images indicate the fluorophore as opposed to the antigen targeting - please correct.

Adding the negative control to Figure 1, instead of only in a supplement, would still be advisable, but not absolutely necessary.

**Answer:** We thank the reviewer for his suggestion. However, we think this is a misunderstanding. Negative control, in this case, means that we incubated the section only with the secondary antibody, omitting the primary antibody, in order to show false positive staining of the secondary antibody. This is the reason why we indicated the fluorophore and not the antigen.

As can be seen in the answer to Reviewer 1, it is impossible to have a TNFR1 negative control using the *TNFRp55d* mice, since they still express a truncated and non-functional TNFR1 protein, that can be detected by the antibody that binds to an amino acid sequence at the C-terminus.

Reviewer #3: (No Response)

Reviewer #4: The authors have tried to address all the comments to the best of their abilities. However, I still have several concerns:
**Q1.** My primary issue is the quality of the immunofluorescence data. The quality of the mouse eye sections (they look battered) are still quite terrible and in good conscience cannot allow this to be published. The VEGFA stain is quite peculiar. A schematic to show the location of the suture would be helpful with the limbus and parts of the eye clearly labeled so one may interpret authors images.

**Answer:** The quality of the mouse section depends on the processing of the sections. Embedding in paraffin, sectioning, rehydrating and especially the heat mediated antigen retrieval can lead to separations between the different corneal layers, which are very thin in the mouse cornea. Nonetheless, the various corneal layers, epithelium, stroma and endothelium, can be differentiated and are displayed very well in our figures. Indeed, the labeling is not sufficient; therefore we named the anatomic structures and marked the position of the sutures in our Figure 1, Supp. Figure 1 and 4. The limbus region is of particular interest due to the outgrowth of blood and lymph vessels into the cornea to the sutures.

**Q2.** Please show data points for all graphs.

**Answer:** We agree with the reviewer about the importance of showing all data that is included in the experiments to ensure and improve scientific transparency and reproducibility. In our opinion, however, including all data points in the individual graphs would lead to graphs that are very confusing and difficult to interpret.  All data points and all raw data analyzed for this manuscript are available at the open access database Zenodo using the following link (<https://doi.org/10.5281/zenodo.4049178>). If Reviewer 3 is still convinced that the manuscript might profit from adding all data points, we can change the graphs accordingly.

**Q3.** The images on the pdf are still terrible. The quality of the fig page improves when downloaded. It is quite onerous for the reviewer to download each image (especially under the current pandemic situation and working from home). I don't know why the supplementary images are not part of the document. Please do so in the next iteration.

**Answer:** We are very sorry for the bad quality of the pdf generated by the submission website. Unfortunately, we are not able to change this. When you download the images, they are in an adequate resolution and quality.

The journal`s guidelines for submissions said to submit the supplement separately. We followed this suggestion. We add the images to the rebuttal letter to ease the review process.

**S1 Fig.** Isotype controls for the secondary antibodies applied in Figure 1 and Figures 1B,C and S2 Fig, respectively. **A** (left side) depicts epithelium and stromal part of the cornea, while **A** (right side) depicts stroma and endothelium. Scale bars represent 100µm (**A**) or 50µm (**B**).

**S2 Fig.** Immunohistochemical staining of corneal sections of sutured wild-type, *TNF-Rp55d*, and *TNF-Rp75d* mice in the suture and the limbus area using antibodies against VEGF-A (green) and CD68 (red). Nuclei were stained with DAPI. Scale bar represents 50 µm.

**S3 Fig. uncropped WB of TNFa (Fig4) A, B:** Uncropped representative images of Western blots used for the densitometric analysis in Fig4C. All uncropped Western blots are shown in the Data repository.
